# Supplementary material for: Prevalence and correlates of microalbuminuria in high-risk persons with hypertension, diabetes, or HIV at a tertiary hospital in Zambia
Source: PLoS One. 2025 Jul 14;20(7):e0328529. doi: 10.1371/journal.pone.0328529 (PMC12258559; doi:10.1371/journal.pone.0328529)
Supplement: Attachment — Submitted filename: response to reviewer file.pdf [file pone.0328529.s003.pdf]

Mulungushi University School of Medicine and Health Sciences
HAND Research Group
P.O Box 60009, Livingstone Campus.
Email:smasenga@mu.ac.zm website:www.mu.ac.zm Contacts: +260 966674774

03.01.2025

To the Editor,

Dear Editor,

Ref: Response to reviewer: PONE-D-24-60503 research article titled ***"Prevalence and Correlates of Microalbuminuria in High-Risk persons"***

Reviewer:
The ethics documentation provided does not appear to provide approval for the study described. It is, therefore, unclear whether you obtained the necessary ethical approval for the study. As a result of these concerns we cannot consider the manuscript for publication at PLOS ONE. I am sorry that we cannot be more positive on this occasion but hope that you understand the reasons for this decision.

Response:

Thank you for your response. Please kindly take note that this manuscript is one of the aims of the project with the ethical approval letter provided "IMPACT OF CLINICAL LABORATORY TEST PROFILE ON THE CLINICAL MANAGEMENT AND OUTCOME OF ACUTE KIDNEY INJURY AT LIVINGSTONE UNIVERSITY TEACHING HOSPITAL "ICLATA STUDY".

I understand that you could not link our submission to the approved project. We have therefore taken the liberty to **attach the actual proposal submitted to ethics** as **"other file".** Kindly review aim 2 to confirm that the study is part of the ICLATA study. The main study is still under review and we could not reference it. However, we have included this write up in the methods ethics section in the resubmitted manuscript.

We have also included a letter from ethics clarifying the issue.

We hope this clarifies your doubts. As researchers we continue to uphold all ethical standards and as an editorial member of PLOS One, meeting ethical requirements for human subjects research submissions and all applicable standards for the ethics of experimentation and research integrity (http://journals.plos.org/plosone/s/human-subjects-research) IS MY PRIORITY.

Yours sincerely,

Prof. Sepiso K. Masenga

Editorial member/editor: PLOS ONE
